# Impact of the 2009 (7th Edition) AJCC Melanoma Staging System in the Classification of Thin Cutaneous Melanomas

**DOI:** 10.1155/2013/898719

**Published:** 2013-12-03

**Authors:** Vicki H. Chu, Michael T. Tetzlaff, Carlos A. Torres-Cabala, Victor G. Prieto, Roland Bassett, Jeffrey E. Gershenwald, Michael S. McLemore, Doina Ivan, Wei-Lien (Billy) Wang, Merrick I. Ross, Jonathan L. Curry

**Affiliations:** ^1^Department of Pathology, Baylor College of Medicine, Houston, TX 77030, USA; ^2^Section of Dermatopathology, Department of Pathology, The University of Texas MD Anderson Cancer Center, Houston, TX 77030, USA; ^3^Department of Biostatistics, The University of Texas MD Anderson Cancer Center, Houston, TX 77030, USA; ^4^Department of Surgical Oncology, The University of Texas MD Anderson Cancer Center, Houston, TX 77030, USA

## Abstract

*Context*. The 7th (2009) edition of the AJCC melanoma staging system incorporates tumor (Breslow) thickness, MR, and ulceration in stratifying T1 primary melanomas. Compared to the prior 6th (2001) edition, MR has replaced CL for thin melanomas. *Objective*. We sought to identify and report differences of the classification of thin melanomas as well as outcome of SLNB in patients according to the 6th and 7th editions at our institution. *Results*. 106 patients were identified with thin melanomas verified by wide excision. 31 of 106 thin melanomas were reclassified according to the 7th edition of the AJCC. Of those 31, 15 CL II/III patients (6th edition T1a) were reclassified as T1b based on the presence of mitoses while 16 CL IV patients (6th edition T1b) were categorized as T1a based on the absence of mitoses. 26/31 reclassified patients underwent SLNB, and all were negative. Patients with thin melanoma and a +SLNB (*N* = 3) were all classified as T1b according to both staging systems. *Conclusions*. In our experience, 29% of thin melanomas were reclassified according to the 7th edition with similar proportions of patients re-distributed as T1a (14%) and T1b (15%). Cases with +SLN corresponded with T1b lesions in both 6th and 7th editions.

## 1. Introduction

Primary cutaneous melanoma is a serious type of skin malignancy and accounts for the majority of skin cancer deaths. The incidence of cutaneous melanoma has risen steadily and in the United States alone has increased at an annual rate of 3.1% over the past several decades [1]. The estimated lifetime risk of an American developing invasive melanoma is 1 in 59 and is projected to rise to 1 in 50 by the year 2015 [2]. Increased surveillance and enhanced public awareness have contributed to early detection of thin melanomas and have likely contributed to the increased incidence of melanoma [3]. The incidence rates for both thick and thin melanoma continue to increase for individuals 65 years or older and [4] thin melanomas account for 30% of total melanoma deaths. These features underscore the biologic significance of these tumors and the importance of wide excision and the need to delineate the subset of thin melanomas with the capacity for a more aggressive clinical course [4].

The tumor-node-metastasis (TNM) classification of melanoma defines the stage of disease and guides patient management decisions. The staging and prognosis of cutaneous melanoma continue to be refined; the most recent version was published as 2009 (7th edition) AJCC melanoma staging system. Breslow tumor thickness (BT) is one of the most important predictors of prognosis for invasive primary cutaneous melanoma [5–8]. Historically, the depth of tumor invasion has been described in terms of both Clark level (CL) and BT. CL defines the anatomic compartment of the skin involved by melanoma cells, whereas BT represents the maximum vertical thickness of the tumor measured from the top of the granular cell layer to the deepest invasive component [9, 10]. Thin melanomas as defined by the American Joint Committee on Cancer (AJCC) melanoma staging system are invasive lesions with BT ≤ 1.0 mm [7]. Thin melanomas account for approximately 70% of cutaneous melanoma diagnoses and are overall associated with a favorable prognosis [11, 12]. The ten-year melanoma specific survival for thin melanomas ranges from 97 to 82% depending on BT, mitosis, and ulceration [13]. Therefore, a subset of thin melanomas has a risk for metastasis and death [7, 14].

Historically, both CL and BT were used to define T categories, while primary tumor ulceration was incorporated into the staging system later [6]. Tumor ulceration is a poor prognostic indicator, and ulcerated melanomas were upstaged beginning with AJCC revisions in 2001. The 2001 AJCC melanoma staging system used CL and ulceration to subclassify thin melanomas as T1a or T1b. Primary tumors with CL IV or V or with ulceration were staged as T1b whereas lesions with CL < IV and no ulceration were staged as T1a [6]. Analyses culminating in the 7th edition of the AJCC melanoma staging database identified primary tumor BT, ulceration, and mitotic rate (MR) as the dominant independent predictors of survival in the histopathologic stratification of thin cutaneous melanomas [7, 13]. Significantly, MR was determined to be a stronger predictor of melanoma specific survival of thin melanomas when compared with CL; therefore, MR has replaced CL in the subclassification of T1 melanomas according to the 2009 AJCC staging and classification system [7, 15–17]. The presence of primary tumor ulceration continues to define T1b melanomas [7].

Regional nodal tumor burden is another important prognostic factor of survival for patients with invasive cutaneous melanoma. Lymphatic mapping and sentinel lymph node biopsy (SLNB) facilitate the evaluation and identification of nodal disease [18–20]. The sentinel lymph node(s) (SLN) is the first lymph node(s) to receive lymphatic drainage from a tumor at a particular anatomic area [21]. Thus, that SLN is the most likely regional lymph node to contain a metastatic deposit and functions as the best surrogate of nodal disease burden. Overall, the incidence of SLN metastasis for thin melanomas is typically low; however, there is a subset of thin melanomas that demonstrates at least a 5% risk for microscopic nodal disease [22].

We sought to identify a group of patients whose melanomas were evaluated after the implementation of 2009 AJCC staging system and examine the impact of the revised melanoma staging system in stratifying T1a and T1b lesions in patients with confirmed thin melanomas verified by examination of wide excision specimens. The outcome of SLNB, when performed, was also collected among this subset of patients with thin melanomas.

## 2. Material and Methods

The surgical pathology database at a major cancer center was searched for invasive cutaneous melanomas from January 1, 2010, to June 30, 2011. This study was approved by the institutions review board. Patients with biopsies were reviewed by at least one of the members of the dermatopathology faculty (MTT, VGP, CAT, MSM, DI, WLW, and JLC) and included all reported histologic parameters in a melanoma pathology template, specifically including Clark level (CL), Breslow thickness (BT), mitotic rate (MR), and ulceration (U). In addition, SLN status was collected in a subset of patients with verified thin melanomas who had this procedure as part of their management.

Mitotic rate was assessed in the invasive melanoma by examination of hematoxylin and eosin (H&E) stained sections. The number of sections varied depending on whether they were specimens processed at MDACC or were referred from other institutions. At any rate, the evaluation included all sections of routine H&E levels that were available. Histologically acceptable mitotic figures included tumors with condensed chromatin in metaphase, anaphase, or telophase of the mitotic cell cycle. The initial mitotic count, reported as number of mitotic figures/mm^2^, began in the area of invasive tumor with the highest number of mitotic figures or mitotic “hot spot” and examination of successive microscopic fields (4.5 h.p.f at 400x magnification in our Olympus BH-50) until one mm^2^ of tumor had been examined. In thin melanomas with less than one mm^2^ area of invasive tumor, the presence of at least one mitotic figure in any field (i.e., hot spot) constituted a mitotic rate of 1/mm^2^ [8, 23, 24]. Of the 327 patients identified with invasive cutaneous melanoma, 138 patients had reported BT ≤ 1.0 mm. Of these, 106 patients had wide excision available for review and confirmed BT ≤ 1.0 mm. Patients without wide excision verification of initial biopsy of thin melanomas (*N* = 21) and patients with residual tumors with BT > 1.0 mm in wide excision specimens (*N* = 11) were excluded from the final analysis. Patients with residual tumor and CL or BT greater than initially reported were used in the final analysis as long as each reported BT ≤ 1.0 mm. Mitotic rate reported as <1 mitosis/mm^2^ or “not applicable” (i.e., due to a very small dermal component) was considered to be equivalent to 0/mm^2^. Thin melanomas verified by examination of wide excision were classified as T1a and T1b according to both the 2001 and 2009 AJCC staging systems.

A number of patients with wide excision-verified T1 lesions also underwent SLNB. The protocol for histologic evaluation of SLNB at MDACC involved initial examination of H&E stained sections of SLN bread-loafed perpendicular to the long axis and has been described elsewhere [25, 26]. If the SLN was negative on the initial H&E section, a second H&E section (approximately 200 microns deeper into the tissue block) and an additional section submitted for immunohistochemical analysis with panmelanocytic cocktail (includes antibodies to detect HMB45, Mart-1, and tyrosinase) were reviewed to evaluate for the presence of SLN metastasis [26, 27].

Continuous variables of BT and number of mitoses/mm^2^ were compared among groups by using the Wilcoxon rank-sum test. Categorical variables of regression and the presence of an associated nevus were compared between groups by using Fisher exact test. No adjustment was made for the multiplicity of testing.

## 3. Results

Classification of thin melanomas among group of patients whose melanomas were evaluated after the modification of 2009 AJCC melanoma staging systems was compared with 2001 version and is presented in Table 1. The male to female ratio was 1.2 : 1, with age ranging from 11 to 86 years (median 56.5 years). The median BT of each group ranged from 0.30 to 0.90 mm. A total of 31/106 (29%) T1 lesions were reclassified when compared to the 2001 and 2009 AJCC staging guidelines. Sixteen patients with nonulcerated tumors that were CL IV and no mitotic figures were reclassified from T1b in the 2001 AJCC melanoma staging system to T1a according to the 2009 AJCC melanoma staging system. In addition, fifteen nonulcerated T1 lesions with CL II (*N* = 3) or III (*N* = 12) that were defined as T1a under the 2001 melanoma staging system were reclassified to T1b based on the presence of at least one mitotic figure/mm^2^ according to the 2009 AJCC melanoma staging system. Forty-five (42.5%) and 30 (28.3%) of the 106 thin melanomas retained their classification as T1a and T1b lesions according to both 2001 and 2009 AJCC melanoma staging systems, respectively. Overall, a net increase of a single T1a patient (with a commensurate decrease in 1 T1b patient) was noted when the melanomas were reclassified according to the 2009 AJCC melanoma staging system among these patients.

Thin melanomas were further classified according to CL, number of mitoses/mm^2^, ulceration, and, among those patients who had the procedure, status of SLNB. The median BT of 0.30 mm in T1a lesions with CL II, absence of mitosis, ulceration, and negative SLNB was significantly lower (*P* < 0.01)^†^ when compared to all other reclassified patients groups. In the 31 patients whose T-stage was reclassified the median BT ranged from 0.55 mm to 0.79 mm and was not significantly different between the sixteen patients (CL IV, no mitotic figures, no ulceration) previously categorized as T1b and reclassified to T1a when compared to the fifteen patients (CL II or III, with any mitotic rate, no ulceration) previously categorized as T1a and now re-classified as T1b based on the presence of at least one mitosis/mm^2^. The median mitotic rates of the reclassified T1b patients with CL II (*N* = 3) and CL III (*N* = 12) were each 1/mm^2^. The category with the greatest median BT (0.90 mm) was CL III with MR > 1 and ulceration (*N* = 3). This group also had the highest median mitotic rate of 3/mm^2^; however, SLNB were all negative (0/3). Tumor ulceration was detected in 3.8% (4/106) of the T1 lesions. All patients with ulcerated tumors (*N* = 4) had SLNB, and all of these were negative (0/4).

The three patients with positive SLNB were classified as T1b under both the 2001 and 2009 AJCC melanoma staging systems and had a median BT of 0.81 mm and a median MR of 2/mm^2^. All three patients had CL IV tumors with absence of ulceration and regression and no associated nevus. Deep margin involvement on initial biopsy exam was absent in this cohort. In this cohort of T1b patients with positive SLNB, the average BT and MR were not significantly different when compared to the 15 reclassified T1b patients with negative SLNB.

**Figure 1:**
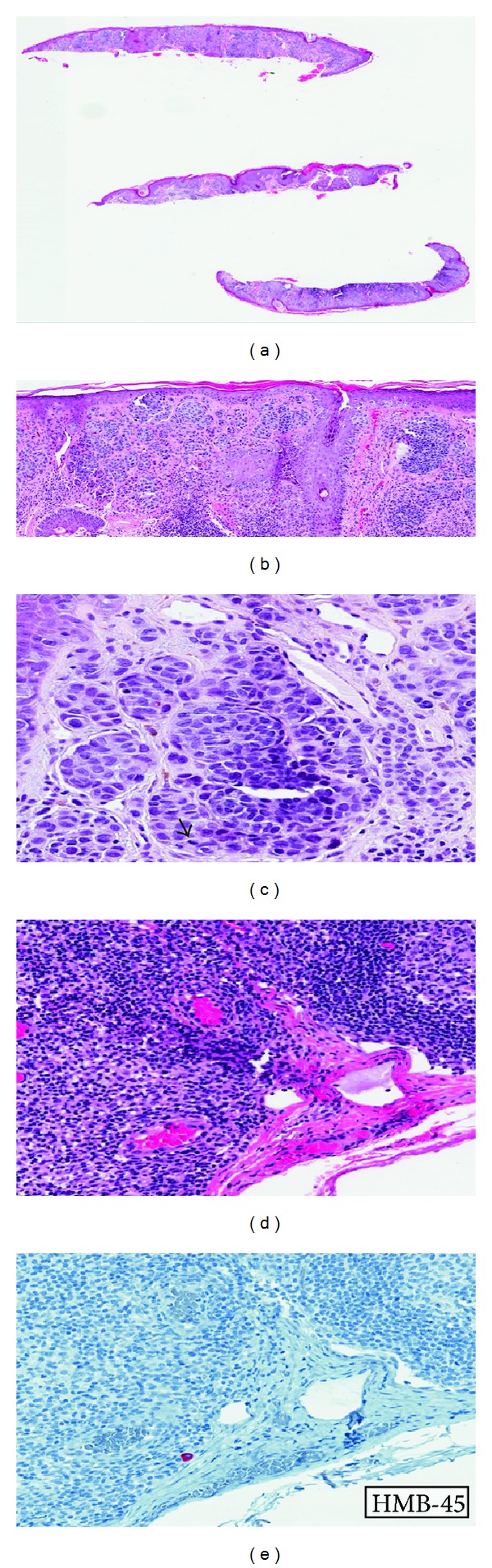
Representative case of thin melanoma ((a), (b)) with dermal mitosis ((c), arrow) and corresponding SLNB (d) with isolated HMB45+ melanoma cell (e) hematoxylin-eosin stain; original magnifications: ×1 (a); ×20 (b); ×40 ((c), (d), (e)).

The clinical and histopathologic characteristics of these three patients with positive SLNB are shown in Table 2 and a representative example of thin melanoma and positive SLNB in Figure 1. Tumor infiltrating lymphocyte (TIL) response, satellitosis, and perineural invasion (PNI) were also considered for analysis; however, nearly all measurements were categorized as nonbrisk (TIL) and without satellitosis or PNI. Thus, these parameters were not subsequently analyzed. The presence of regression or an associated nevus between groups was not statistically significant. Each positive SLNB case demonstrated microscopic foci of tumor with <10 cells detected by immunohistochemistry only (Figure 1). Completion lymph node dissections (CLND) were negative in all of these patients.

The percent of SLNB performed for thin melanomas is shown in Table 3. SLNB was performed in 61.3% (65/106) of patients with thin melanomas in this cohort. Of these, 26.2% (17/65) were lesions designated with the descriptor of “at least” regarding BT, since tumor cells involved the base of the shave biopsy specimen. Patients with T1 melanomas that had the same subclassification under both the 2001 and 2009 AJCC melanoma staging system had SLNB at a rate of 31% for T1a and 87% for T1b. Among the T1b patients, 3 had positive SLNB with microscopic tumor deposits (fewer than 10 tumor cells detected in SLN). 83% (26/31) of patients with reclassified T1a and T1b tumors had SLNB and all were negative. In this study, patients with positive SLNB had BT of at least 0.54 mm, whereas all patients with BT < 0.54 mm had negative SLNB.

## 4. Discussion

Early detection of thin melanomas remains an essential factor for long-term survival since melanomas less than 1.0 mm in thickness have a good prognosis and predicted 10-year survival of up to 97%. While surgical excision may be curative in greater than 90% of patients with thin melanomas (T1 lesions), there is a subset of patients who may ultimately develop aggressive clinical behavior.

Staging of cutaneous melanoma continues to evolve as risk factors are identified to be associated with poor or favorable survival. In analyses leading to its incorporation into the 2009 AJCC melanoma staging system, mitotic rate was determined to be an important histologic parameter in evaluating thin melanomas. Mitotic rate was an independent adverse predictor of survival and ultimately replaced Clark level to define T1b lesions [7, 13, 15]. MR ≥ 1/mm^2^ and BT > 0.76 mm are worrisome histological parameters for micrometastasis in a subset of patients with thin melanomas; however, the criteria for selecting “high-risk” thin melanomas for SLNB continue to evolve [28–31].

Mitotic rate is determined by the number of mitoses per millimeter squared (mm^2^) in the invasive melanoma component and all sections on the slides should be examined to evaluate for mitoses or “hot spot.” In thin melanomas that comprise less than a one mm^2^ (or less than 4.5 h.p.f at 400x magnification) area of invasive tumor available for examination, the presence of mitoses in any field (hot spot) is constituted as a mitotic rate ≥1/mm^2^. MR should not be averaged or reported as a fraction (e.g. 0.5/mm^2^), and mitoses of intraepidermal melanocytes should not be included in the MR. Standard clinical practice is to interpret MR of <1/mm^2^ or “not applicable” due to small dermal component as MR = 0/mm^2^. Additional levels and/or depletion of tissue block in search for mitoses should be avoided.

This study was conducted to evaluate the differences in the distribution of T1a and T1b melanomas according to the 2001 and 2009 AJCC melanoma staging system. In our study, 29% of the T1 melanomas were reclassified. According to the 2009 AJCC melanoma staging system, there were a greater number of T1a melanomas (61/106 or 57.5%) compared with T1b melanomas (45/106 or 42.4%); however, the percentage of T1a and T1b melanomas was similar by both 2001 and 2009 AJCC melanoma staging system. SLNB was performed in 83.9% (26/31) of patients reclassified as T1a and T1b—all of which had negative SLNB. In our study, the 2009 AJCC melanoma staging system did not identify additional T1b thin melanomas with positive SLNB that would have been classified as T1a under the 2001 staging system. All patients who had a positive SLNB were classified as T1b under both the 2001 and 2009 AJCC melanoma staging systems.

The risk of SLN metastasis for thin melanomas has been reported to vary from 1.1% to 16.0% [22, 32]. Historically (i.e., prior to the 6th edition AJCC staging system), “thin melanomas” were defined as tumors with BT < 0.75 mm. In one study, thin melanomas with BT ≥ 0.75 mm had a greater than 5.0% risk for SLN metastasis, while melanomas <0.75 mm had a 2.7% risk for SLN metastasis [33]. Another study reported a progressive increase in rates of positive SLNB for invasive melanomas depending on BT: 0.51 to 0.75 mm (3.8%), 0.76 to 0.90 mm (5.3%), and 0.91 to 1.00 mm (10.3%) [34]. Patients with BT < 0.5 mm in the study by Murali et al. did not have any positive SLNB (*N* = 37) [34]. In our study, SLNB was performed on 61.3% (65/106) of the thin melanomas examined and was positive in 4.6% (3/65) of patients. If we applied BT < 0.50 mm as a cutoff point as in the study by Murali et al., in our cohort patients with thin melanomas with BT < 0.50 mm had no positive SLNB (0/15). In contrast, patients with thin melanomas of BT ≥ 0.50 mm had a 6.0% (3/50) rate of positive SLNB.

All patients in this study with SLN micrometastasis lacked regression or an associated nevus. This is consistent with contemporary studies demonstrating that regression is not a predictor of a positive SLNB in thin melanomas [35–37]. Previously, the presence of regression was considered to be a risk factor for SLN metastasis; however, based on currently available data, standard clinical practice does not generally incorporate regression as a criterion for SLNB in patients with thin melanomas [8, 37–39].

However, it should be noted that a study by Guitart et al. demonstrated that a subset of thin melanomas with extensive regression (defined as regression in 50% or more of the lesion) poses a risk for metastasis [40]. Further studies are needed to determine if the extent of regression (defined as a percentage of the lesion) correlates with prognosis or SLN status.

## 5. Conclusion

This study corroborates the importance of MR in thin melanomas as a risk factor for SLN metastasis and the role of SLNB in a subset of patients with thin melanomas [16, 22, 34, 41, 42]. All patients with positive SLNB were classified as T1b according to 2001 and 2009 staging systems. While it is evident that a subset of thin melanomas behaves aggressively with some risk for distant metastasis and death from melanoma, the clinical-pathologic parameters for SLNB in patients with thin melanomas continue to evolve [7, 14, 43]. In particular, CL IV invasion in thin melanomas has been described by some authors to pose a risk for SLN metastasis [41, 44–47], while other studies have demonstrated CL IV invasion not to be predictive of SLN involvement in tumors when compared to BT and ulceration [48, 49]. Previous studies have shown by univariate analysis clinical-pathologic factors such as BT, CL, MR, ulceration status, anatomic site, age, and gender as significant predictors of nodal metastasis in thin melanomas [47, 50]. However, by multivariate analysis, only BT, gender, and age remained significant for thin lesions [43, 47]. Factors to be considered for SLNB in patients with thin melanomas among at least some melanoma experts include patients with BT ≥ 0.76, MR ≥ 1/mm^2^, primary tumor ulceration, “young” age, and superficially shaved lesions with positive deep margins which may underestimate the actual BT [28, 31, 34, 44, 47, 49, 51]. However, ultimately the selection of a subset of patients with thin melanomas for SLNB requires coordinated discussion between the patient and clinician.

This is a retrospective study and is limited by a small sample size (*N* = 106) of patients with thin primary melanomas; of note, only 3 patients in this cohort had a positive SLNB. Furthermore, a selection bias towards more aggressive disease may be introduced in our patient sample since our institution is a referral center for high-risk patients. To further address significant clinical-pathological features that define a subset of patients with higher risk thin melanomas we intend to examine a larger cohort of thin cutaneous melanomas.

Continued reporting of all the histologic parameters as previously described for all (including thin) melanomas will permit incorporation of all primary tumor characteristics into clinical management and facilitate continued collection of data to further assess “high-risk” clinical-pathologic factors in patients with thin melanomas [52].

## Figures and Tables

**Table 1:** Classification of patients with T1 invasive melanoma according to the 2001 and 2009 AJCC staging and classification system (*N* = 106).

Melanoma categories			2001 AJCC, 6th edition		2009 AJCC, 7th edition		Reclassified T1 melanomas according to 2009 AJCC staging	Median BT in mm (range)
Clark level	Mitosis	Ulceration	T1a	T1b	T1a	T1b		
II	−	−	31	0	31	0	No	0.30^†^ (0.12–1.0)
II	**+**	**−**	**3**	**0**	**0**	**3**	**Yes**	**0.70** (**0.44–0.80**)
II	−	+	0	1	0	1	No	0.64 (0.64–0.64)
II	+	+	0	0	0	0	No	—
III	−	−	14	0	14	0	No	0.55 (0.38–0.85)
III	**+**	**−**	**12**	**0**	**0**	**12**	**Yes**	**0.79** (**0.35–1.0**)
III	−	+	0	0	0	0	No	—
III	+	+	0	3	0	3	No	0.90 (0.65–0.94)
IV	**−**	**−**	**0**	**16**	**16**	**0**	**Yes**	**0.55** (**0.32–0.98**)
IV*	+	−	0	26	0	26	No	0.70 (0.44–0.98)
IV	−	+	0	0	0	0	No	—
IV	+	+	0	0	0	0	No	—

Total			60	46	61	45	31	0.60 (0.12–1.0)

Reclassified T1a and T1b cohorts are listed in bold. Median BT of 0.30 mm was significantly lower compared to BT in other categories^†^: (*P* < 0.01). Mitosis “+”: 1 or more dermal tumor mitosis/mm^2^; mitosis “−”: <1 or 0 dermal tumor mitosis/mm^2^; ulceration “+”: histological absence of epidermis with accompanying inflammatory crust; ulceration “−”: histologically intact epidermis; BT: Breslow thickness; *category with positive sentinel lymph node biopsy (SLNB).

**Table 2:** Clinical and histopathologic characteristics of the three T1 patients with positive SLN.

Age	Gender	Location	Histologic type	CL	BT (mm)	MR	Positive SLN	Total no. SLN	No. of tumor cells in SLN	No. of positive LN/CLND
34	M	Postauricular	Superficial spreading	IV	0.81	3	2	4	<10	0/45
53	M	Midabdomen	Superficial spreading	IV	0.81	1	1	1	<10	0/15
68	M	Arm	Nodular	IV	0.54	2	1	1	<10	0/22

CL: Clark level; BT: Breslow thickness; MR: mitotic rate; TIL: tumor infiltrating lymphocytes; Reg: regression; LVI: lymphovascular invasion; PNI: perineural invasion; Sat: satellitosis; SLN: sentinel lymph node; no.: number; LN: lymph node; CLND: completion lymph node dissection; NB: nonbrisk; NI: not identified.

**Table 3:** Staging and rate of SLNB in reported cohorts of patients with thin melanomas.

	No. of patients	Median age in years (range)	CL	Median BT in mm (range)	No. of patients with SLNB (%)	Patients with + SLNB	No. of positive SLN/total no. SLN
BT < 0.5 mm	39	55 (21–85)	II–IV	0.32 (0.12–0.49)	15 (38)	0	0/37
BT ≥ 0.5 mm	67	59 (11–86)	II–IV	0.70 (0.50–1.0)	50 (75)	3	4/139

Total	106	56.5 (11–86)	II–IV	0.60 (0.12–1.0)	65 (61)	3	4/176

CL: Clark level; BT: Breslow thickness; SLNB: sentinel lymph node biopsy; SLN: sentinel lymph node; no.: number.
